# Mechanical Properties and Uniaxial Failure Behavior of Concrete with Different Solid Waste Coarse Aggregates

**DOI:** 10.3390/ma15186259

**Published:** 2022-09-08

**Authors:** Mei Zhou, Jinting Bai, Shaowei Li, Kai Zhang, Chao Li, Xinyi Wang

**Affiliations:** 1 College of Civil Engineering, Liaoning Technical University, Fuxin 123000, China; 2 Liaoning Provincial Key Laboratory of Coal Gangue Resource Utilization and Energy-Saving Building Materials, Fuxin 123000, China; 3 Fujian Building Research Institute Co., Ltd., Fuzhou 350025, China; 4 Fujian Provincial Key Laboratory of Green Building Technology, Fuzhou 350025, China

**Keywords:** solid waste coarse aggregate, concrete, mechanical properties, elastic modulus, stress–strain curve, microstructure

## Abstract

To reveal the differences between the mechanical properties of solid waste coarse aggregate concrete and natural coarse aggregate concrete (NCAC) under equal strength, the basic mechanical properties of coarse aggregate concrete with seven different solid wastes (i.e., self-combusted coal gangue, uncombusted coal gangue, marble sheet waste, granite sheet waste, iron waste rock, recycled concrete, and self-combusted coal gangue ceramicite) were tested, and the trends in failure morphology, elastic modulus, and the stress–strain full curves of the different solid waste coarse aggregate concretes were analyzed and compared with NCAC. Finally, the interfacial structure of the concrete was characterized by SEM. The results showed that C30 strength grade concrete was prepared with different solid waste coarse aggregates; however, the 28 d compressive strength, split tensile strength, axial compression strength, flexural strength, and elastic modulus of the concrete was 35.26–47.35, 2.13–3.35, 26.43–42.70, 2.83–3.94, and 17.3–31.2, respectively. The modulus of elasticity of the solid waste coarse aggregate concrete was smaller than the NCAC under equal strength, with a maximum difference of 45%. The peak compressive strain and ultimate compressive strain were larger than the NCAC, with a maximum difference of 43%. The crushing value of the solid waste coarse aggregate affected the splitting tensile strength, flexural strength, and modulus of elasticity of the concrete to a greater extent than the compressive strength. The transition zone at the concrete interface of the coarse aggregates with different solid wastes varied widely. The porous micro-pumping effect of the self-combusted gangue and self-combusted gangue vitrified reinforced the concrete interface transition zone, and the polished surface of sheet waste, uncombusted gangue, and recycled concrete aggregate surface adhesion weakened the interface transition zone; Finally, the uniaxial compressive stress–strain curve model for concrete with different solid waste coarse aggregates was established based on the Guo Zhenhai model.

## 1. Introduction

China is the largest developing country in the world, but the rapid development and industrialization has undermined the harmonious development of human beings and the environment. With various mining, energy, heavy industry, chemical industry, civil engineering, and other industries as representatives, more than 5 billion tons of solid waste are generated each year, accounting for more than 2 million hectares, and the total amount of solid waste accumulated over the years exceeds 60 billion tons [1,2]. The massive output of solid waste is related to the extensive mode of production and outdated technology over the past few decades. More than 80% of the ore mined becomes tailings, which not only occupies valuable land resources but also brings serious risks to the environment and safety [3,4]. With the continuous aggravation of resource and environmental constraints in China, a trend toward carrying out a multipath comprehensive utilization of solid waste resources under the guidance of “carbon peaking and carbon neutrality” is inevitable. On the other hand, with the increase in infrastructure investment, the shortage of aggregates for concrete is becoming increasingly prominent. The main elements, such as oxygen, silicon, aluminum, iron, calcium, sodium, and potassium, in solid waste from the mining industry account for more than 90%, and their chemical and mineral compositions are similar to those of natural stone. Some solid waste contains a small number of harmful components, which can also be used as aggregates for concrete production after slight treatment in order to realize the recycling of solid waste [5]. The application of solid waste aggregate concrete, not only to solve the problem of solid waste disposal but also to reduce the ecological damage caused by the overexploitation of resources, therefore has a broad development prospects.

In recent years, research enthusiasm for “solid waste” as aggregates of concrete has continuously improved at home and abroad. For example, YuxiangTang et al. [6] proposed and discussed an empirical stress–strain model using recycled coarse and recycled fine aggregates to completely replace natural aggregates and recycled powder (RP) to partially replace cement based on test results. Liu et al. [7] studied the mechanical properties and damage evolution behavior of coal-fired slag concrete (CSC) specimens under uniaxial compressive loading, and they determined the damage evolution process of CSC. The CSC can be divided into three stages: an initial damage formation stage, a damage-accelerated growth stage, and a damage failure stage. Bai et al. [8] conducted uniaxial compression tests on recycled aggregate concrete by varying the water–cement ratio and the replacement rate of recycled aggregates, and the results showed that the four characteristic parameters, ε_a_, ε_h_, ε_b_, and H, had obvious regularity with the increase in the replacement ratio. It was suggested to take the critical state as the final damage state of the intrinsic model to fully consider the ductility of the material distribution damage stage and to avoid the size effect of the local damage stage. Yun [9] et al. used glass waste as coarse particles in concrete in combination with superplasticizers to improve its strength, and they determined and optimized the optimum amount of glass waste as a substitute for coarse aggregates. Markssuel Teixeira Marvila et al., presented relevant aspects of materials [10] that could be applied in the production of HPC and UHPC, as well as prospects for new materials that could be considered in the production of new special concrete. Deng Z [11] et al. investigated the effect of recycled coarse aggregate classification on the mechanical behavior of recycled aggregate concrete (RAC) using recycled coarse aggregate and substitution rate as factors. Ostrowski K. [12] studied the potential use of granite waste as coarse aggregate in high-performance and self-compacting concrete. Thomas C. [13] studied the mechanics and durability of concrete, partially replacing natural fine aggregate with granite cutting waste, and Vardhan K. [14] studied the influence of marble waste as fine aggregate on concrete performance. Machi A. E. [15] studied the preparation of concrete, which fully replaced gravel with the waste rock produced by phosphate mining. Blessen Skariah Thomas [16] studied the strength and durability of concrete instead of natural aggregate by the aggregate of copper tailings. Karimipour A. [17] studied the performance of coal gangue aggregate concrete with different contents under fiber reinforcement, and Li [18,19] studied light aggregate concrete and recycled concrete with marine clay ceramsite. Zhu [20] and Zhang [21] studied the feasibility and durability of concrete with unburned coal gangue aggregate, and Zhou [22,23,24] and Wang [25] carried out a series of studies on the spontaneous combustion of coal gangue aggregate concrete. Xiao [26,27] and Wang [28,29] carried out many basic and applied studies on recycled concrete, and the results showed that “solid waste”, as aggregate partially or completely replacing natural aggregate to prepare medium- and low-strength concrete, is feasible [30,31,32,33,34,35,36]. In summary, although many studies have been conducted on solid waste coarse aggregate and solid waste coarse aggregate concrete, but most of these studies focused on the effect of a particular solid waste coarse aggregate replacement rate on the mechanical properties of concrete, and lacked a systematic comparative analysis of concrete formulated with a 100% replacement of natural coarse aggregate with different solid waste coarse aggregates. Solid waste aggregates are generally multicomponent mixed aggregates with different particle sizes, surface morphology, and crushing values, which led to the extreme complexity of the concrete structure formation process and the large differences with ordinary concrete [37]. The traditional model of the principal structural relationship of ordinary concrete can no longer guide the application, and it is necessary to carry out mechanical property tests on solid waste coarse aggregate concretes, such as compressive, splitting tensile, flexural, axial compression, modulus of elasticity, and stress–strain, to propose a new microstructure and principal structure relationship to meet the needs of the structural analysis and the design of solid waste aggregate concrete and to better guide the engineering application.

In this paper, combined with the current ranking of the accumulated amount of solid waste in China, based on an analysis of the different physical and chemical properties of solid waste, natural coarse aggregate (NCA), such as limestone gravel, was taken as the baseline control group, and seven solid wastes were selected as follows: self-combusted coal gangue ceramicite (SCC), self-combusted coal gangue (SCG), unburned coal gangue (UCG), recycled concrete aggregate (RCA), iron waste rock (IWR), marble sheet waste (MSW), and granite sheet waste (GSW). They were all siliceous aggregates. The continuous-graded coarse aggregate, with a size of 5–20 mm, was prepared using the same process, which replaced 100% of the natural aggregates to prepare the C30 concrete, respectively, and the C30 concrete was prepared by completely replacing the NCA. Through the mechanical properties test and the microscopic characterization of 168 specimens with different sizes and eight ratios, the effects of the surface morphology and the crushing value of the solid waste coarse aggregate on the compressive strength, splitting tensile strength, flexural strength, axial compressive strength, elastic modulus, and compressive stress–strain curve of the solid waste coarse aggregate concrete were studied. The constitutive relationship between the microstructural characteristics and macroscopic behavior of the different solid waste coarse aggregate concretes was revealed, and the constitutive model of the concrete considering the characteristic parameters of the solid waste coarse aggregate was established, which provides theoretical guidance and technical support for the application of solid waste coarse aggregate concrete in engineering.

## 2. Materials and Methods

### 2.1. Physicochemical and Mechanical Properties of Different Solid Waste Coarse Aggregates

**Figure 1:**
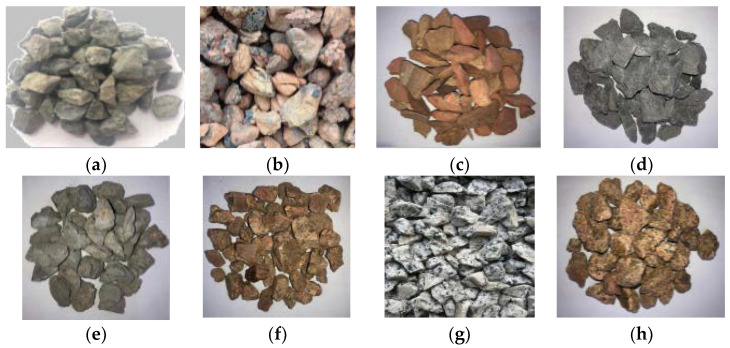
Appearance of the different coarse aggregates: (**a**) NCA; (**b**) SCC; (**c**) SCG; (**d**) UCG; (**e**) RCA; (**f**) IWR; (**g**) MSW; (**h**) GSW.

**Figure 2:**
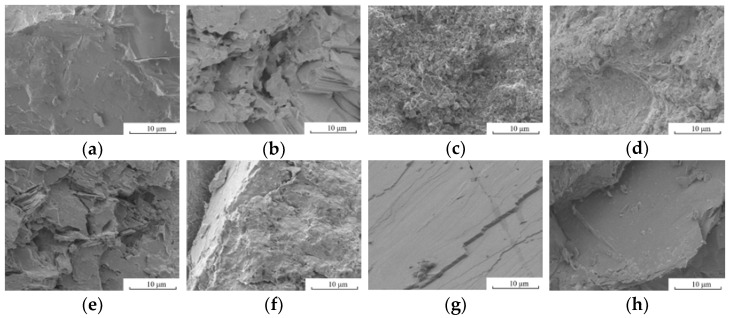
Microstructure of the different coarse aggregates: (**a**) NCA; (**b**) SCC; (**c**) SCG; (**d**) UCG; (**e**) RCA; (**f**) IWR; (**g**) MSW; (**h**) GSW.

**Figure 3:**
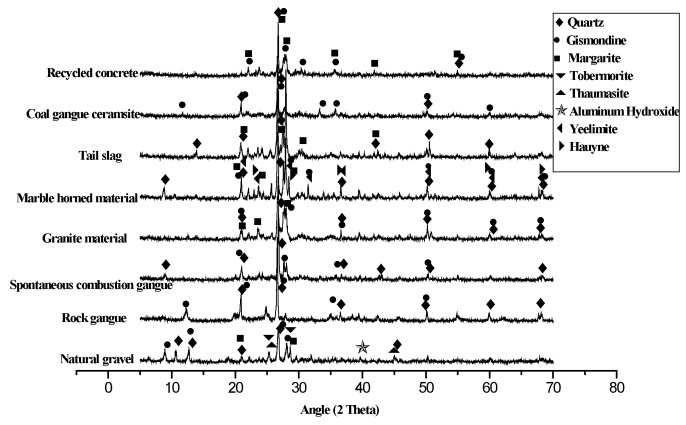
XRD of the different coarse aggregates.

Firstly, the raw solid waste was prepared into different sizes of single-graded coarse aggregate by jaw crushing → screening, and other preparation processes, and then graded according to “Recycled Coarse Aggregate for Concrete” (GB/T25177-2010) and, finally, coarse aggregates with a particle size of 5–20 mm with a continuous gradation was obtained, and the particle size and gradation of the eight different coarse aggregates were almost the same. The macro- and micromorphologies of the seven kinds of solid coarse aggregates are shown in Figure 1 and Figure 2, and their XRD patterns are shown in Figure 3. According to the “Standard for technical requirements and test method of sand and crushed stone for ordinary concrete” (JGJ 52-2006), the physicochemical and mechanical properties of the above coarse aggregates were tested, and the results are shown in Table 1. According to “Recycled Coarse Aggregate for Concrete” (GB/T 5177-2010) and other standards, the quality of the different kinds of solid waste coarse aggregate was comprehensively evaluated.

#### 2.1.1. Appearance

It can clearly be seen from Figure 1 and Figure 2 that the macro- and micromorphologies of the different coarse aggregates were quite different. According to the different classifications of the aggregate compositions, the coarse aggregate could be divided into single-component aggregate and multicomponent aggregate. In terms of the surface, it was mostly pottery red, with some light yellow or dark red; the SCC and SCG were the multicomponent aggregates. The internal porosity of the SCC and SCG was large, the material homogeneity was poor, and the content of the needle-like particles was high. The UCG and RCA were also multicomponent aggregates with obvious adhesion on the surface, poor homogeneity, and high content of needle-like particles. The other four coarse aggregates were single-component aggregates with good uniformity and grain shape, but there were some polished surfaces on the coarse aggregates of the MSW and GSW. From Figure 3, it can be seen that the main mineral compositions of the eight different coarse aggregates were quartz, gismondine, a small amount of margarine, etc. The different solid waste aggregates selected in this paper were all siliceous aggregates.

#### 2.1.2. Bulk Density, Apparent Density, and Porosity

It can be seen from Table 1 that the coarse aggregate of SCC met the of a lightweight aggregate with a density grade of 1000. It can be judged from the apparent density (ρ_0_) and porosity (P_0_) that the coarse aggregates of the NCA, UCG, IWR, MSW, and GSW met the requirements of class I (ρ_0_ > 2450 kg/m^3^; P_0′_ < 47%), the coarse aggregate of the RCA met the requirements of class II (ρ_0_ > 2350 kg/m^3^; P_0′_ < 50%), and the coarse aggregate of the SCG met the requirements of class III (ρ_0_ > 2250 kg/m^3^; P_0′_ < 53%).

#### 2.1.3. Water Absorption

The water absorption (W_m_) of the NCA, IWR, MSW, and GSW was less than 3%, which met the requirements of class I (≤3%). The W_m_ of the SCC and UCG was less than 5%, which met the requirements of class II (≤5%). The W_m_ of the SCG and RCA was less than 8%, which met the requirements of class III (≤8%).

#### 2.1.4. Crushing Value

The crushing values (Qa) of the NCA, UCG, and IWR were less than 10%, which met the requirements of class I (≤10%). The Qa of the RCA, MSW, and GSW was less than 20%, which met the requirements of class II (≤20%). The Qa of the SCC and SCG was less than 30%, which met the requirements of class III (≤30%).

In conclusion, the seven solid waste coarse aggregates selected in this experiment had different granular shapes, apparent densities, water absorption, crushing values, and mineral compositions, and they were significantly different from the NCA in the technical properties, but they still met the requirements of the different classes of coarse aggregates in GB/T 5177-2010.

### 2.2. Other Materials

The binding materials were cement (P·O 42.5R), fly ash (F-II), and slag (S95), and the main technical properties and chemical constituents are shown in Table 2 and Table 3. The fineness modulus of the fine aggregate grading zone II from the local river was 3.16. In addition to the solid waste coarse aggregates, introduced in Section 2.1, the natural coarse aggregate was continuously graded limestone gravel from 5 to 20 mm. The test water was from tap water. The water-reducing agent was polycarboxylate superplasticizer, the dosage of which was from 1.5% to 2.5%, and the water reduction rate was from 20% to 30%.

### 2.3. Design

Natural aggregate concrete (NCAC) formulated with natural sand and stone was used as the reference group, and a total of eight concrete proportions with different coarse aggregates, mix slump of 30 to 50 mm, and strength grade of C30 was designed. There were three groups of cubic specimens with molding sizes of 100 × 100 × 100 mm, two groups of prismatic specimens with a size of 100 × 100 × 300 mm, and one group of prismatic specimens with a size of 100 × 100 × 400 mm, and a total of 18 specimens in each mixing ratio and 144 specimens with eight mix ratios. In order to test the accuracy of the prediction model, another four groups of 12 prismatic specimens with sizes of 100 × 100 × 300 mm and 12 cubic specimens with sizes of 100 × 100 × 100 mm were formed for a total of 168 specimens.

According to “Specification for mix proportion design of ordinary concrete” (JGJ 55-2011) and “Technical specification for lightweight aggregate concrete” (JGJ 51-2002), the test mix proportion of the concrete was calculated and finally determined, as shown in Table 4, after the adjustment of the initial mix ratio, the basic mix ratio, and the mix ratio of laboratory and construction. Combined with the characteristics of large porosity and water absorption of the SCC, SCG, and RCA, three kinds of solid waste coarse aggregates were humidified with additional water in advance to ensure the construction requirements of the concrete mixture.

### 2.4. Test Method

**Figure 4:**
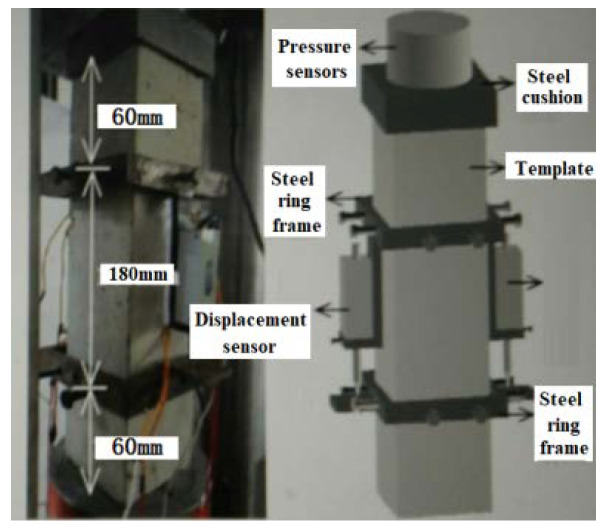
Picture of the stress–strain loading concrete.

**Figure 5:**
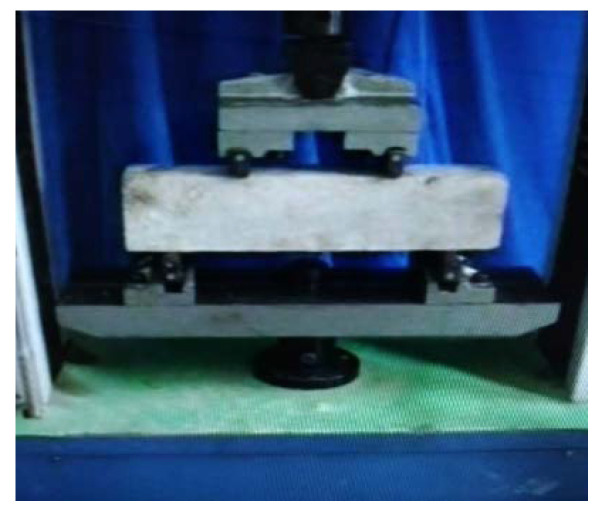
Picture of the flexural loading concrete.

All specimens were maintained in a standard curing room until the specified ages, and the mechanical tests were carried out in accordance with the relevant test methods in the “Standard for test methods of concrete physical and mechanical properties” (GB/T 50081-2019). Cubic specimens of 100 × 100 × 100 mm were used for tests on the compressive and splitting tensile strength, prismatic specimens of 100 × 100 × 300 mm were used for the axial compressive strength, elastic modulus, and stress–strain curve, and prismatic specimens of 100 × 100 × 400 mm were used for the flexural strength. The cubic and axial compression tests were carried out on the electro-hydraulic servo universal testing machine (1000 kN), using a displacement control loading system, and the loading rate was controlled at 0.01 mm/s. The dynamic data acquisition system, strain gauge, and extensometer were used to collect the test data in the stress–strain test of the concrete, and the complete stress–strain curve under compression was plotted. The splitting tensile and flexural tests were carried out on an electro-hydraulic servo universal testing machine (300 kN). The loading mode of the tests is shown in Figure 4 and Figure 5.

A scanning electron microscope (SEM) was used on the above 28 day old specimens. After the cube compressive strength test, the interface between the cement paste and aggregate was selected from the fragments, and the observation surface of the samples was required to be as flat, small, and thin as possible, of which the maximum did not exceed 1 cm^3^. After being pumped to the vacuum state, the surface was sprayed and placed under SEM. The surface morphology of the samples was observed by JSM-7500F in a cold field (accelerated voltage: 5 kV).

## 3. Results and Discussion

### 3.1. Mechanical Properties

The mechanical properties of the concrete with different coarse aggregates are shown in Table 5 (the test data in Table 5 are the average values of three specimens).

The cubic compressive strength (f_cu_), splitting tensile strength (f_ts_), axial compressive strength (f_cp_), flexural strength (f_tm_), and elastic modulus (E) of the concrete with solid waste coarse aggregates were taken as the average values of three specimens. It can be seen from Table 5 that the compressive strength of the different kinds of solid waste coarse aggregate concrete met the design requirements of C30. The order of f_cu_ and f_cp_ of the concrete with seven kinds of coarse aggregates was IWRC > MSWC > GSWC > RCAC > NCAC > UCGC > SCCC > SCGC. The order of f_ts_ was NCAC > GSWC > IWRC > MSWC > SCGC > RCAC > UCAC > SCCC. The order of f_tm_ was NCAC > IWRC > GSWC > MSWC > RCAC > SCGC > IWRC > UCGC. The axial compression ratio (f_cp_/f_cu_), tension–compression ratio (f_ts_/f_cu_), and compression ratio (f_tm_/f_cu_) of the seven solid waste coarse aggregate concretes were 0.74–0.93, 0.062–0.091, and 0.079–0.108, respectively. It can be observed from Table 5 that there was no positive correlation between the compressive strength and elastic modulus of the seven solid waste coarse aggregate concretes. Combining Table 1 and Table 5, it was found that the relationship between the crushing value of the solid waste coarse aggregates and the compressive strength of the concrete was not positive, but the crushing value had a negatively correlated impact on the splitting tensile and flexural strength of the concrete. The SCC, SCG, and RCA with a higher water absorption had a significant effect on the compressive strength of the concrete. Appropriate consumption of additional water can ensure the initial slump and time loss of the concrete mixture meets the construction requirements, and the SCC and SCG with a “micro pump” and pozzolanic reaction were beneficial to the later strength development of the concrete. The compressive strength of the RCAC was lower, because (a) the high water absorption rate of the RCA reduced the effective water–binder ratio, resulting in inadequate hydration of the cement; (b) vibration made the water absorbed by the attached slurry move to the new interface, leading to “bleeding” and making the interface loose and porous; (c) as the water–binder ratio of the new local interface increased, more Ca(OH)_2_ was formed by hydration and enriched on the surface of the attached slurry.

### 3.2. Failure Characteristics

**Figure 6:**
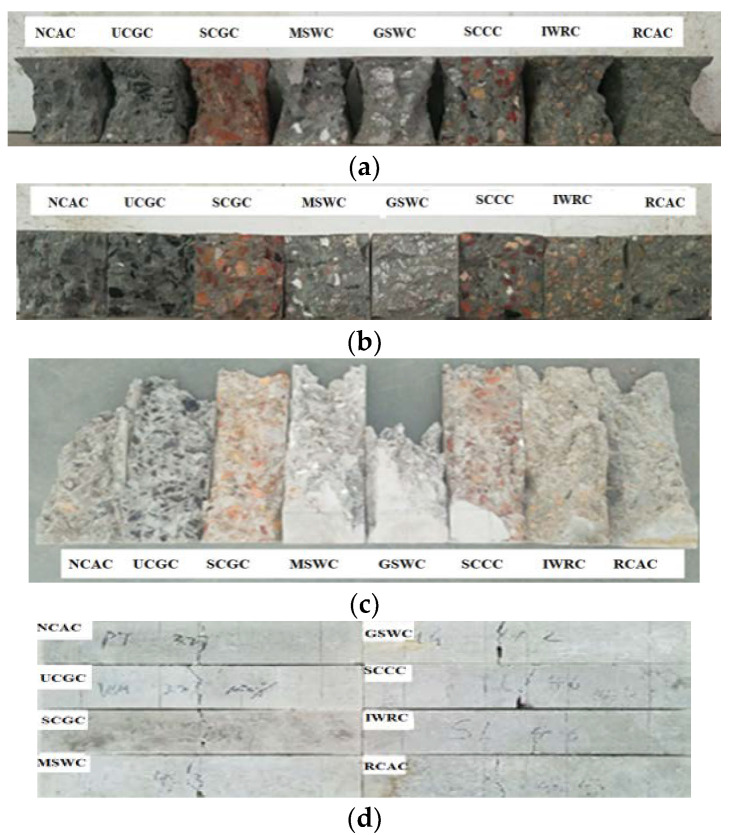
Failure characteristics of different kinds of coarse aggregate concrete: (**a**) cubic compression; (**b**) splitting tension; (**c**) axial compression; (**d**) bending.

The failure characteristics of cube compression, split tension, axial compression, and flexural specimens are shown in Figure 6.

The cubic compression failure process and morphology of the concrete with solid waste coarse aggregates were different but similar to that of the NCAC. The first short and thin visible vertical cracks appeared from the side of the specimen when the load was 40% to 70% of the ultimate load. With a further increase in the load, the cracks expanded from both sides and ended in the middle, gradually forming a “X” shape with an angle of approximately 45°, which was quickly penetrated and destroyed. The cracks in the MSWC, GSWC, IWRC, RCAC, and NCAC expanded steadily, while the failure process of the SCCC and SCGC was sudden and almost lost the bearing capacity at the same time as the crack occurred, accompanied by a splitting sound. The fracture of the main failure surface of the specimens was neat as shown in Figure 6a. It can be seen from Figure 6b that (a) the coarse aggregates on the splitting surfaces of the NCAC, IWRC, MSWC, and GSWC were relatively intact, and only a small number of coarse aggregates with a poor grain shape were broken; (b) the MSW and GSW were taken from the cutting waste of the factory, and there were some polished surfaces on the coarse aggregates, which basically occurred at the failures of the MSWC and GSWC at the interface between the coarse aggregates and the cement paste; (c) the failure surface passed through the coarse aggregate, and it can be seen that the SCC and SCG were split or cut from the fracture; (d) the coarse aggregates of the UCGC and RCAC on the splitting surfaces had fractures and pull-outs.

There were no visible cracks in the prismatic specimens at the initial loading stage, and a small number of vertical microcracks appeared in the middle of the specimens when the load was close to the peak stress. After reaching the peak stress, as the strain continues to increase, microcracks continued to develop and connect, forming oblique cracks and, finally, cracks ran through the whole specimen. From Figure 6c, the failure characteristics of NCAC, UCGC, GSWC, MSWC, IWRC, and RCAC under axial compression exhibited obvious shear failure in the oblique section, while the failure of the SCGC and SCCC was characterized by a longitudinal splitting failure. From Figure 6d, with typical failure characteristics of brittle materials, the flexural failure morphology of the solid waste coarse aggregate concrete was similar to that of ordinary concrete. Because of the same properties of other raw materials and the same particle size of coarse aggregates in this experiment, the different failure processes and characteristics of concrete were mainly caused by the technical properties of the coarse aggregates.

### 3.3. Brittleness Analysis

It can be seen from Table 5 that the f_ts_/f_cu_ and f_tm_/f_cu_ of the solid waste coarse aggregate concretes were inferior to that of the NCAC, indicating that the ductility of the solid waste coarse aggregate concretes was worse than that of the NCAC. The mechanical properties of concrete were related to the types, proportions, and material properties of the raw materials. It can be seen from Table 4 that the differences in the mechanical properties of the different kinds of solid waste coarse aggregate concretes in this paper mainly came from the crushing value, water absorption rate, and surface characteristics of the coarse aggregates. Because of the different lithology, preparation, and processing of the solid waste, there were some coarse aggregates with large internal damage and more microcracks, some with poor surface cleanliness and grain shape and some with poor homogeneity composed of different lithologies, which makes solid waste coarse aggregate concretes more brittle than the NCAC with a compound effect. Therefore, it is suggested to improve the toughness of solid waste coarse aggregate concretes by adding functional materials, such as fiber, and strengthening the surface of the aggregates.

### 3.4. Microstructure Analysis

**Figure 7:**
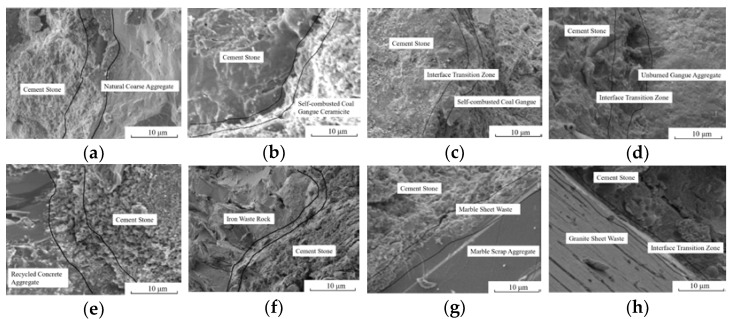
SEM of different kinds of coarse aggregate concrete: (**a**) NCAC; (**b**) SCCC; (**c**) SCGC; (**d**) UCGC; (**e**) RCAC; (**f**) IWRC; (**g**) MSWC; (**h**) GSWC.

The microstructural characteristics and performance of the concrete largely determined the macroscopic performance of the concrete. The microscopic morphology of concrete with different solid waste coarse aggregates is shown in Figure 7.

The surface characteristics of solid waste coarse aggregates do affect the microstructure of the interfacial transition zone. From Figure 7a, there is a loose and coarse pore structure in the interfacial transition zone of NCAC, where crystalline products have sufficient space for growth and development, but the interfacial bonding is not tight enough. Because NCAC has the largest water-cement ratio and the lowest amount of binding material among the eight mixing ratios, and denser NCA, the interface has a large water-cement ratio and the weakest part of the NCAC, and the damage to concrete is mostly caused by NCA’s displacement at the interface. The results of this study are consistent with those of NEMATI K M [37]; From Figure 7b,c, the interfacial transition zone of SCCC and SCGC is fuzzy, which means dense in structure and good performance in interfacial bonding. The reasons are as follows: (a) The water–cement ratio of the SCCC and SCGC was not much different from that of the NCAC, but the porous light aggregates of the SCC and SCG had the effect of a “micro pump”, which made them absorb water and reduce the water–cement ratio at the interface to improve the interface strength in the initial stage. In the late stage, the hydration of cement is fully guaranteed by releasing water from SCC and SCG, to reduce the pore channel at the interface and improve the pore structure. (b) The elastic modulus of the SCC and SCG was similar to that of set cement, which made the deformation of the coarse aggregate and set cement tend to be consistent and reduced the defects in the interfacial area. (c) Some cement particles hydrate inside the aggregate pore, which combines the hydration products outside the pore to produce an interfacial transition zone with no obvious boundary and makes the set cement firmly “grasp” the coarse aggregates, the interfacial performance significantly improved. The above findings coincide with the findings of [38]. (d) Because SCG has pozzolanic properties, fine powder attached to the SCC and SCG participates in a hydration reaction under the excitation of Ca(OH)_2_. However, due to the poor porosity and homogeneity of coarse aggregates, many places in the SCCC and SCGC were pulled off after loading, which means that the quality of the coarse aggregate is the key to affecting the strength of the concrete.

It can be seen from Figure 7d,e that there were adhesions on the surface of UCG and RCA, which leads to an obvious interfacial transition zone between coarse aggregate and new mortar. The interfacial transition zone is loose and porous, which was the weakest area in the UCGC and RCAC and affected the strength improvement of the UCGC and RCAC. This finding is in agreement with the findings of the literature [22,25,39,40,41]; From Figure 7f–h, cutting waste of MSW and GSW have a large number of polished surfaces on the coarse aggregate to smooth the interfacial transition zone, which will be harmful to the strength of concrete. The microcracks in the interfacial transition zone rapidly expand along the aggregate interface under compressive load, which shows that the interfacial transition zone of MSWC and GSWC is the weakest area, and the failure mode of concrete is almost the pull-out from the coarse aggregate interface. From Figure 7g, it can be seen that the coarse aggregate/slurry interface has a large amount of Ca(OH)_2_ enrichment and forms an obvious directional arrangement, which is in agreement with the results of the study of Ref. [37]. The IWR aggregate was obtained by crushing iron tailing ore, and it can be seen from Table 4 that the water–cement ratio of this group was the smallest, and there was no obvious transition zone between cementite, aggregate, and the interface, and the three-phase matching of the cement matrix, coarse aggregate, and interface were high; thus, the compressive strength of concrete formulated by IWRC was the highest.

### 3.5. Complete Stress–Strain Curve

#### 3.5.1. Influence of Solid Waste Coarse Aggregates on the Complete Stress–Strain Curve

**Figure 8:**
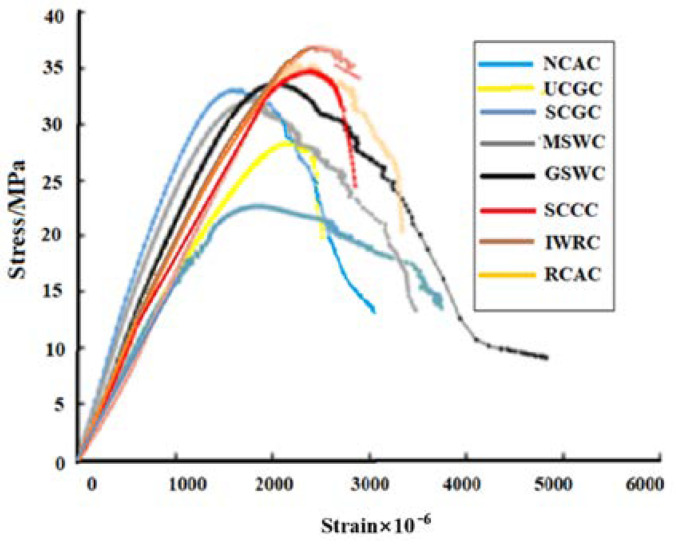
Complete stress–strain curve of the different kinds of the coarse aggregate concretes.

The complete stress-strain curve of concrete under uniaxial compression is the most basic constitutive relation needed in structural design. It can be seen from Figure 8 that (a) the ascending part of the complete stress–strain curve of the different kinds of solid waste coarse aggregate concrete became slower than that of the NCAC, and the slope of the ascending part was smaller than that of the NCAC, indicating that the elastic modulus was smaller than that of the NCAC, which was mainly related to the failure mode of the solid waste coarse aggregate concrete. (b) Compared with the NCAC, SCGC, and SCCC had steeper stress–strain curves and smaller areas under the curves, indicating that the ductility of the SCGC and SCCC was inferior to that of the NCAC. The reason for this is that the porous lightweight aggregates of the SCG and SCC had larger deformations under stress but had lower compressive and split-tensile strength, and the SCGC and SCCC had a larger peak strain and smaller elastic modulus. However, the splitting of the SCG and SCC made the cracks extend quickly, with a large slope and small number, resulting in a sharp failure process of SCGC and SCCC and a rapid decline in the residual strength. (c) The stress–strain curve of the solid waste coarse aggregate concrete was flatter than that of the NCAC, and the peak strain and ultimate strain were greater than those of the NCAC with the same strength grade. The reason for this is that there were many microcracks in the waste coarse aggregate of its own porous or in the process of production or formation, which makes the deformation larger, resulting in a larger peak strain and lower elastic modulus than the NCAC.

It can be seen from Figure 8 that only the NCAC and GSWC had obvious back-bending and convergence points in the falling section of the stress–strain curve, and no obvious back bending and convergence points can be seen in the falling section curves of the remaining six different solid waste coarse aggregate concretes, which may be related to the properties of the solid waste coarse aggregates. After the concrete reached peak stress cracks continue to expand and penetrate, resulting in more and more serious damage to the internal structure, and gradually losing the load-bearing capacity. At this time, the development of cracks formed in the specimens accelerated, NCAC and GSWC concrete cracks are cement mortar crack penetration, and coarse aggregate to prevent crack penetration. The crack penetration in the remaining six types of solid waste concrete is not only the cement mortar, probably solid waste coarse aggregate (the solid waste in the production or aggregate in the preparation process under the action of external forces produce more microscopic cracks inside) also involved in the penetration.

#### 3.5.2. Stress–Strain Curve models of Solid Waste Coarse Aggregate Concrete

**Figure 9:**
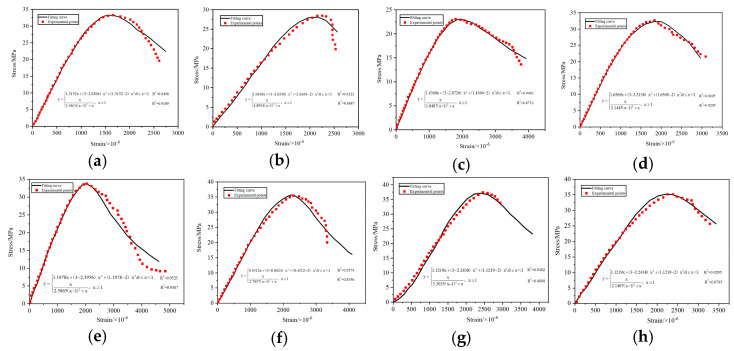
Stress–strain curve models of different kinds of coarse aggregate concrete: (**a**) NCAC; (**b**) UCGC; (**c**) SCGC; (**d**) MSWC; (**e**) GSWC; (**f**) IWRC; (**g**) SCCC; (**h**) RCAC.

Professor Guo [41,42] believes that stress-strain curves of all kinds of concrete can be expressed by Equation (1), which is written in “Code for the design of concrete structures” (GB 50010-2010). In this paper, based on this two-stage model and the experimental data in Figure 8, given the small amount of data in this study and the data collected in the relevant literature [12,21,23,27,43], finally, the least squares method was used to calculate the fit and obtain the intrinsic parameters of the compressive stress-strain curves for concrete with different solid waste coarse aggregates, which az shown in Figure 9.

(1) $$y=\left\{\begin{array}{l} \mathrm{ax}+\left(3-2a\right)x^{2}+({a-2)x}^{3},0\leq x<1\\ \frac{x}{b(x-1{)}^{2}+x},x\geq 1 \end{array}\right.$$

where x—ε/ε_p_; y—σ/σ_p_; “a” is the shape parameter of ascending section; “b” is the shape parameter of descending section.

It can be clearly seen from Figure 9 that although the full compressive stress-strain curves of different solid waste coarse aggregate concrete are different, the theoretical model curves and the test curves are in good agreement based on the concrete principal structure equations fitted by the Guo Zhenhai model, especially the compressive stress-strain rising section curves are smoothly connected, the transition is natural, and the degree of agreement is high, and the locations where the peak compressive strain and ultimate compressive strain appear were in good agreement with the test values. From Figure 9, it can also be seen that the descending section of the curve is more consistent in the early stage, and the error increases significantly in the later stage. The main reason is as follows: it is well known that the strain energy accumulated in the test machine is released with the decrease of the stress in the descending section of the concrete stress–strain curve. The sudden recovery deformation of the test machine is enough to break the concrete specimen with serious damage to the internal structure. Although the jack auxiliary device is installed on both sides of the specimen during the test, the reason is that first of all, the strain rate operation of the descending section may be wrong; secondly, the strain of descending segment is mainly collected by extensometer, and the precision of extensometer (10^−3^ mm) is far less than that of strain gauge (10^−6^ mm) of ascending section. Third, due to the lack of data in the descending section, the dispersion was large. Therefore, the error of the descending section of the concrete stress–strain relationship curve is large. A lot of work still needs to be done on the parameters of the descending section in the future. However, as long as sufficient information is available, satisfactory results can be obtained through statistical analysis.

In summary, the full stress–strain curves of concrete with different solid waste coarse aggregates can explain the failure characteristics of concrete with different solid waste coarse aggregates more accurately, and the establishment of the principal structure models of different solid waste coarse aggregates can be applied to finite element analysis, which is also the basis for structural strength calculation, section ductility analysis, and structural internal force analysis.

In order to test the accuracy of the fitting equation, three representative groups of NCAC, UCGC, and SCGC were selected, and six specimens of each group were formed and tested for the uniaxial compressive stress–strain curve of concrete after standard curing for 28 d. The test results showed that the error of NCAC is 4.83%, the error of UCGC is 8.92%, and the error of SCGC is 6.17%, which indicates that the modified Guo Zhenhai compressive stress–strain full curve prediction model has a better fit for both rising and falling sections and is recommended to be used.

### 3.6. Modulus of Elasticity of Concrete with Solid Waste Coarse Aggregates

The elastic modulus of concrete is a necessary parameter to calculate the stress, deformation, and crack of structure and is an important index of engineering material performance. It can be seen from Table 5 that the elastic modulus of solid waste coarse aggregate concrete is smaller than that of the NCAC. The reasons are as follows: (a) Compared with single-component aggregates of NCA, MSW, GSW, and IWR, the elastic modulus of concrete prepared by multicomponent mixed aggregates (mixtures with different lithologies), such as UCG, RCA, SCC, and SCG, varied greatly in different regions. At the same time, the large difference in water absorption of the multi-component mixed aggregates leads to the uneven distribution of pores in concrete, which will reduce the elastic modulus of concrete. (b) MSW, GSW, and IWR were derived from natural stone, but MSW and GSW were from the cutting waste, which led to the internal damage and microcracks of aggregates caused by the processing. In addition, there were a large number of polished surfaces in the coarse aggregates which affect the bonding with the cement mortar matrix. In mineral processing, IWR has internal micro-cracks caused by mechanical or chemical damage, which decreases the elastic modulus of IWRC. (c) The porosity of coarse aggregate determines its stiffness, which controls the ability of coarse aggregates to constrain the strain of set cement [29]. Due to the large porosity of SCC and SCG, the stiffness is weakened, which has a great influence on the elastic modulus of SCCC and SCGC. In addition, high water absorption of coarse aggregate will increase the opening porosity of concrete, which will lead to an increase in the decreased elastic modulus of concrete. (d) In addition to the poor homogeneity of UCG and RCA, there are adhesions on the surface, which affects the bonding between coarse aggregates and cement mortar matrix, resulting in the decrease of the elastic modulus of concrete.

As mentioned above, most of the solid waste coarse aggregates had complex components, fluctuating quality, poor mechanical properties and stability, and other defects, leading to different solid waste coarse aggregate concrete moduli of elasticity less than the NCAC.

## 4. Conclusions

(1) Although the different solid waste coarse aggregate concrete met the design requirements of the C30 concrete, the uniaxial failure process and morphology were different. Most of the coarse aggregates fractured in the failure sections of SCCC and SCGC, while they partially fractured in the UCGC and RCAC. The failure characteristics of GSWC, MSWC, and IWRC were similar to those of the NCAC, and the failure was mostly bonding failure between the coarse aggregate and cement mortar;
(2) The uniaxial compressive stress–strain full curves of different solid waste coarse aggregate concrete have different shapes, and the slope of the rising section of the uniaxial compressive stress–strain full curve is smaller than that of NCAC, so the modulus of elasticity of different solid waste coarse aggregate concrete is smaller than that of NCAC. The effect of technical properties of solid waste coarse aggregates on the descending part is more obvious than that on the ascending part, and the peak compressive strain and ultimate compressive strain were both larger than those of the NCAC. All seven different solid waste coarse aggregate concrete damage forms showed typical brittle damage characteristics;
(3) The micro-pumping of SCC and SCG mad the concrete have a better structure in the interfacial transition zone, but it was insufficient to compensate for the decrease in concrete strength caused by the breakage of the SCC and SCG. Because of the polished surfaces of the MSW and GSW, and the interfacial adhesions of the RCA and UCG, the interfacial transition zone was still the weakest area in the concrete under the present test conditions. For solid waste coarse aggregate concrete, the structure of the interfacial transition zone was key to its mechanical properties;
(4) As a more mature principal structure model of ordinary concrete, the Guo Zhenhai model is still applicable to the description of the compressive stress–strain curve of solid waste coarse aggregate concrete. The compressive stress–strain curve of the solid waste coarse aggregate concrete was greatly influenced by the crushing value of solid waste coarse aggregate, pore structure, surface characteristics, etc. The establishment of a uniaxial compressive stress–strain full curve model of the different solid waste coarse aggregate concrete can provide a reference for the calculation analysis and design of different solid waste coarse aggregate concrete structures.

## Figures and Tables

**Table 1:** Physical and mechanical properties of the different coarse aggregates.

	Indexes	Apparent Density/kg·m^−3^	Bulk Density/kg·m^−3^		Porosity/%	1 h Water Absorption/%	Crushing Value/%
Variety			Loose	Tight			
NCA		2505	1470	1660	41.32	1.55	4.50
SCC		1817	993	1105	45.35	3.20	25.84
SCG		2276	1075	1220	46.39	7.98	20.80
UCG		2630	1340	1489	46.91	2.15	9.90
RCA		2524	1334	1809	38.66	5.80	18.24
IWR		2375	1334	1520	43.83	1.10	9.05
MSW		2620	1450	1590	44.66	0.46	11.78
GSW		2743	1520	1680	44.58	0.43	11.18

**Table 2:** Main technical properties of the cement.

Compression Strength/MPa		Flexural Strength/Mpa		Normal Consistency Water Demand/%	Time of Setting/Min		Volume Stability
3 d	28 d	3 d	28 d		Initial Set	Final Set	
27.1	51.8	5.1	8.6	25.9	169	259	Qualified

**Table 3:** Main chemical components of the cementitious materials (w%).

	Composition	SiO_2_	Al_2_O_3_	Fe_2_O_3_	CaO	MgO	SO_3_	TiO_2_	K_2_O	Na_2_O
Variety										
Cement		25.84	7.16	2.73	52.79	3.54	2.71	--	1.43	0.91
Fly ash		62.02	11.29	5.19	6.07	1.56	1.04	1.0	1.63	0.31
Slag		35.46	20.33	4.87	20.98	4.52	3.85	--	--	--

**Table 4:** Mix ratio of the different kinds of coarse aggregate concrete (kg/m^3^).

	Material	Cement	Fly Ash	Slag	Mixing Water	Water Reducer	Fine Aggregate	Coarse Aggregate
Type								
NCAC		253	70	95	175	11.17	753	1040
SCCC		340	105	80	210	4.60	463	640
SCGC		334	96	43	205	10.38	632	1031
UCGC		328	94	47	190	8.64	626	1021
RCAC		334	96	43	205	10.38	632	1031
IWRC		328	94	47	168	8.75	626	1021
MSWC		324	93	46	182	4.42	628	1025
GSWC		324	93	46	185	4.63	628	1025

NCAC, SCCC, SCGC, UCGC, RCAC, IWRC, MSWC, and GSWC represent concrete prepared, respectively, with coarse aggregates of NCA, SCC, SCG, UCG, RCA, IWR, MSW, and GSW.

**Table 5:** Mechanical properties of the different kinds of coarse aggregate concrete.

	Index	Cube Compressive Strength/MPa		28 d Split Tensile Strength/MPa	28 d Axial Compressive Strength/MPa	28 d Flexural Strength/MPa	28 d Elastic Modulus/GPa	28 d Tension Compression Ratio	28 d Bending Compression Ratio
Type		7 d	28 d						
NCAC		26.88	36.87	3.35	34.18	3.98	31.2	0.091	0.108
SCCC		17.57	35.26	2.13	33.17	3.44	17.3	0.060	0.099
SCGC		24.28	35.98	2.25	26.43	3.49	17.7	0.075	0.108
UCGC		25.59	35.95	2.39	28.98	2.83	18.4	0.062	0.079
RCAC		26.53	37.09	2.32	34.18	3.27	19.2	0.063	0.088
IWRC		24.64	47.35	2.91	42.70	3.86	20.2	0.062	0.082
MSWC		26.11	41.27	2.78	30.61	3.28	25.8	0.067	0.087
GSWC		26.38	40.49	3.11	31.17	3.64	24.4	0.077	0.090

## Data Availability

Not applicable.

## References

[1] Lian H.Z., Yan P.Y. (2004). Facing challenge of sustainable development to concrete industry. Archit. Technol..

[2] Marvila M., de Matos P., Rodríguez E., Monteiro S.N., de Azevedo A.R.G. (2022). Recycled Aggregate: A Viable Solution for Sustainable Concrete Production. Materials.

[3] Zhang Y., Dong M., Zhang W., Chen H., Yang D. (2022). Preparation of Mineral Admixture from Iron Tailings with Steel Slag-Desulfurization Ash and Its Application to Concrete. Materials.

[4] Gu X.W., Zhang Y.N., Zhang W.F., Zhao Y.Q., Li X.H., Wang H.Y. (2022). Research status and prospect of high value building materials utilization of bulk industrial solid waste. Met. Mine.

[5] Wang D.M. (2020). Carbon neutrality and solid waste resource utilization. Bull. Chin. Ceram. Soc..

[6] Tang Y., Xiao J., Zhang H., Duan Z., Xia B. (2022). Mechanical properties and uniaxial compressive stress-strain behavior of fully recycled aggregate concrete. Constr. Build. Mater..

[7] Liu W., Guo Z., Niu S., Hou J., Zhang F., He C. (2020). Mechanical properties and damage evolution behavior of coal–fired slag concrete under uniaxial compression based on acoustic emission monitoring technology. J. Mater. Res. Technol..

[8] Bai W., Li W., Guan J., Wang J., Yuan C. (2020). Research on the mechanical properties of recycled aggregate concrete under uniaxial compression based on the statistical damage model. Materials.

[9] Yun C.M., Rahman M.R., Kuok K.K., Chai A.P.S., Ding A.B.S., Bin Bakri M.K. (2022). Glass Waste as Coarse Aggregate Filler Replacement in Concrete. Waste Materials in Advanced Sustainable Concrete.

[10] Marvila M.T., de Azevedo A.R.G., de Matos P.R., Monteiro S., Vieira C. (2021). Materials for production of high and ultra-high performance concrete: Review and perspective of possible novel materials. Materials.

[11] Deng Z., Liu B., Ye B., Xiang P. (2020). Mechanical behavior and constitutive relationship of the three types of recycled coarse aggregate concrete based on standard classification. J. Mater. Cycles Waste Manag..

[12] Ostrowski K., Stefaniuk D., Sadowski Ł., Krzywiński K., Gicala M., Różańska M. (2020). Potential use of granite waste sourced from rock processing for the application as coarse aggregate in high-performance self-compacting concrete. Constr. Build. Mater..

[13] Thomas C., Setién J., Polanco J.A. (2016). Structural recycled aggregate concrete made with precast wastes. Constr. Build. Mater..

[14] Vardhan K., Siddique R., Goyal S. (2019). Influence of marble waste as partial replacement of fine aggregates on strength and drying shrinkage of concrete. Constr. Build. Mater..

[15] ElMachi A., Mabroum S., Taha Y., Tagnit-Hamou A., Benzaazoua M., Hakkou R. (2021). Use of flint from phosphate mine waste rocks as an alternative aggregates for concrete. Constr. Build. Mater..

[16] Thomas B.S., Damare A., Gupta R.C. (2013). Strength and durability characteristics of copper tailing concrete. Constr. Build. Mater..

[17] Karimipour A. (2020). Effect of untreated coal waste as fine and coarse aggregates replacement on the properties of steel and polypro pylene fibers reinforced concrete. Mech. Mater..

[18] Li Q.Y., Wang Z.W., Li Y.X., Tian S. (2008). Experimental study on high performance lightweight concrete made from sea mud ceramsite. Mater. Sci. Technol..

[19] Guo Y.X., LI Q.Y., Yue G.B., LI Q.Q. (2018). Calculation of compressive strength of recycled concrete based on coarse aggregate quality and replacement rate. J. Build. Struct..

[20] Zhu Y.Y., Wang A.G., Sun D.S., Liu K.W., Li Y., Chu Y.J. (2021). Characteristics of coal gangue fine aggregates after calcination and its effects on the improvement of mortar properties. J. China Coal Soc..

[21] Zhang J.X., Chen W.L., Jin S.S., Chen C.Z., Yang R.J. (2011). Investigation on durability of coal gangue aggregate Concrete. J. Beijing Univ. Technol..

[22] Zhou M., Dou Y., Zhang Y. (2019). Effects of the variety and content of coal gangue coarse aggregate on the mechanical properties of concrete. Constr. Build. Mater..

[23] LI S.W., Zhou M., Zhang L.M. (2020). Properties of Spontaneous Combustion Coal Gangue Coarse Aggregate and Its Influence on Concrete. J. Build. Mater..

[24] Yang S., Zhou M., Zhang Y.Z., Zhang B., Zhang B.Q. (2020). Effect of spontaneous combustion coal gangue coarse aggregate replacement ratio on fracture properties of three-point bending concrete beam. J. Build. Mater..

[25] Wang Q., Ran K., Wang J.B., Huang Y.Y., Zhang Q. (2021). Study on microstructure characteristics of interface transition zone of spontaneous combustion coal gangue concrete. Concrete.

[26] Tu K., Wu J., Wang Y., Deng H., Zhang R. (2022). Uniaxial Compressive Stress-Strain Relation of Recycled Coarse Aggregate Concrete with Different Carbonation Depths. Materials.

[27] Xiao J.Z., Du J.T. (2008). Complete stress-strain curves of concrete with different recycled coarse aggregates under uniaxial compression. J. Build. Mater..

[28] Wang Q.H., You G.D., Yang J.S., Zhang Y.C., Zhang T.R. (2021). Flexural behavior and design procedures of steel-recycled aggregate concrete composite beams. J. Build. Struct..

[29] Wang Q.H., Liang Y.Z., Zhang H. (2021). Time-dependent behavior of multi-span continuous steel-RAC com positive slabs considering the loading distribution effects. Eng. Mech..

[30] Cong X.Y., Lu S., Yao Y. (2016). Fabrication and characterization of self-ignition coal gangue autoclaved aerated concrete. Mater. Des..

[31] Singh S., Khan S., Khandelwal R. (2016). Performance of sustainable concrete containing granite cutting waste. J. Clean. Prod..

[32] Guo Z., Zhang X., Zhang D., Wang R. (1982). Experimental study of the full stress-strain curve of concrete. J. Build. Struct..

[33] Singh S., Nagar R., Agrawal V. (2016). Performance of granite cutting waste concrete under adverse exposure conditions. J. Clean. Prod..

[34] Chen X.J., Ni W., Wu H., Tang C., Qiu X.J. (2015). Experimental study on high-strength concrete with waste rocks and iron tailing as aggregates. Met. Mine.

[35] Chen Z.P., Zhou C.H., Chen Y.L. (2014). Mechanical properties of recycled pebble aggregate concrete. J. Build. Mater..

[36] Dan J.M., Wang P.M. (2007). Influence of fineness and content of coal gangue on properties of cement pastes. J. Build. Mater..

[37] Dong H., Qian C. (2008). Microstructure of interfacial transition zone of marble-cement and sandstone-cement paste. J. Chin. Ceram. Soc..

[38] Nemati K.M., Paolo G. (2005). Microstructural and Statistical Evaluation of Interfacial Zone Percolation in Concrete, Strength, Fracture, and Complexity.

[39] Guo R., He K., Ma Q., Yan F., Lin Z., Sun Y. (2017). Compressive Properties and Microstructure of Modified Lightweight Aggregate Concrete after Exposure to Elevated Temperatures. J. Chin. Ceram. Soc..

[40] Geng O., Chen C., Gu R., Zhang J., Chai B. (2012). Development Law of Interfacial Microscopic Structure in Recycled Coarse Aggregate Concrete. J. Chin. Ceram. Soc..

[41] Chen Y., Sun Z., Xiao J. (2004). Characteristics and strengthening methods of interfacial zone between aggregate and cement paste in recycled-aggregate concrete. Concrete.

[42] Guo Z.H. (1997). Strength and Deformation Test Foundation and Constitutive Relation of Concrete.

[43] Xiao J.Z. (2007). Experimental Investigation on Complete Stress-Strain Curve of Recycled Concrete Under Uniaxial Loading. J. Tongji Univ..

